# Mid- and long-term changes in satiety-related hormones, lipid and glucose metabolism, and inflammation after a Mediterranean diet intervention with the goal of losing weight: A randomized, clinical trial

**DOI:** 10.3389/fnut.2022.950900

**Published:** 2022-11-18

**Authors:** J Hernando-Redondo, A Toloba, D Benaiges, J Salas-Salvadó, MA Martínez-Gonzalez, D Corella, R Estruch, FJ Tinahones, E Ros, A Goday, O Castañer, M Fitó

**Affiliations:** ^1^Consorcio CIBER, Fisiopatología de la Obesidad y Nutrición (CIBERobn), Instituto de Salud Carlos III, Madrid, Spain; ^2^Unit of Cardiovascular Risk and Nutrition, Hospital del Mar Medical Research Institute, Barcelona, Spain; ^3^Ph.D. Program in Food Science and Nutrition, Universitat de Barcelona, Barcelona, Spain; ^4^Department of Endocrinology, Hospital Universitario del Mar, Barcelona, Spain; ^5^Medicine Department and Life Sciences, Universitat Pompeu Fabra, Barcelona, Spain; ^6^Departament de Bioquímica i Biotecnologia, Universitat Rovira i Virgili, Unitat de Nutrició Humana, Reus, Spain; ^7^Institut d’Investigació Pere Virgili, Hospital Universitari Sant Joan de Reus, Reus, Spain; ^8^Department of Preventive Medicine and Public Health, University of Navarra, Pamplona, Spain; ^9^Department of Nutrition, Harvard T.H. Chan School of Public Health, Boston, MA, United States; ^10^Department of Preventive Medicine, Universidad de Valencia, Valencia, Spain; ^11^August Pi i Sunyer Biomedical Research Institute, Barcelona, Spain; ^12^Internal Medicine Service, Hospital Clinic, Barcelona, Spain; ^13^Department of Endocrinology, Biomedical Research Institute of Málaga, Virgen de la Victoria Hospital, University of Málaga (IBIMA), Málaga, Spain; ^14^Department of Endocrinology and Nutrition, Lipid Clinic, IDIBAPS, Hospital Clínic, Barcelona, Spain

**Keywords:** metabolic syndrome, Mediterranean diet (MedDiet), leptin, PAI-1, inflammation

## Abstract

**Background:**

Obesity is produced by the enlargement of the adipose tissue. Functioning as an endocrine organ, it releases and receives information through a complex network of cytokines, hormones, and substrates contributing to a low-chronic inflammation environment. Diet and healthy habits play key roles in the prevention of obesity and its related pathologies. In this regard, there is a need to switch to healthier and more appetizing diets, such as the Mediterranean one.

**Objective:**

To compare the mid-and long-term effects of two Mediterranean diet (MedDiet) interventions, one energy-reduced plus physical activity promotion versus a non-restrictive diet, on peripheral satiety-related hormones, weight loss, glucose/lipid metabolism, and pro-inflammatory markers in subjects with obesity/overweight and metabolic syndrome.

**Materials and methods:**

A randomized, lifestyle intervention was conducted in 23 Spanish centers, with a large cohort of patients presenting metabolic syndrome. Our study is a subproject set in IMIM (Hospital del Mar Research Institute). Participants were men and women, aged 55–75 and 60–75, respectively, who at baseline met at least three metabolic syndrome components. Subjects were assigned to two intervention groups: (1) an intensive lifestyle intervention with an energy-reduced MedDiet and physical activity promotion (intervention group) with the aim of weight loss; and (2) a normocaloric MedDiet (control). We quantified in a subsample of 300 volunteers from Hospital del Mar Research Institute (Barcelona), following analytes at baseline, 6 months, and 1 year: glucose, HbA1c, triglycerides, total cholesterol, high-density lipoprotein cholesterol, LDL cholesterol, C-peptide, ghrelin, GLP-1, glucagon, insulin, leptin, PAI-1, resistin, and visfatin. Anthropometric and classical cardiovascular risk factors were also determined. A multivariate statistical model was employed to compare the two groups. Linear mixed-effect models were performed to compare changes in risk factors and biomarkers between intervention groups and over time.

**Results:**

Compared to participants in the control group, those in intervention one showed greater improvements in weight, waist circumference, insulin (*P* < 0.001), glucose metabolism-related compounds (*P* < 0.05), triglyceride-related lipid profile (*P* < 0.05), leptin, blood pressure, and pro-inflammatory markers such as PAI-1 (*P* < 0.001) at mid-and/or long-term. High-sensitivity C-reactive protein, resistin, and vifastin also decreased in both groups.

**Conclusion:**

A weight loss intervention employing a hypocaloric MedDiet and physical activity promotion has beneficial effects on adiposity, glucose metabolism, lipid profile, leptin, and pro-inflammatory markers, such as PAI-1 in both mid-and long-term.

## Introduction

Over the past 40 years, obesity has come to be considered an emerging global pandemic. Described by the World Federation of Obesity “as a chronic relapsing disease process,” it has proven influence on the development of hypertension, diabetes mellitus, and cardiovascular events (1). Current nutrition habits, which include the excessive consumption of sweetened beverages and high-density energy food, have notably increased the prevalence of overweight and obesity in both child and adult populations. Moreover, western society has embraced sedentary routines which further contribute to an augmented positive energy balance, thus worsening insulin resistance and perpetuating unhealthy behavioral patterns (2, 3).

Metabolic syndrome, characterized by high cardiovascular risk due to prediabetes/diabetes, hypertension, dyslipidemia, and overweight/obesity, is associated with several comorbidities, including cardiovascular conditions, diabetes, cancer, and liver disease. Specific pharmacological agents apart, there is a need to switch to healthier diets, such as the Mediterranean one (MedDiet), given that diet is a key factor in the prevention of such comorbidities (4).

The traditional MedDiet, largely based on plant-derived products, is characterized by seasonal and proximity products. It includes: (a) olive oil as the main source of fat; (b) high consumption of cereals, vegetables, legumes, fruit, and nuts; (c) moderate intake of poultry, fish, eggs, milk, and dairy products; (d) regular, but moderate, consumption of red wine at meals; and (e) low intake of red meat, processed meat, and industrial confectionery (5). The protective effect of the traditional MedDiet against cardiovascular disease in primary prevention has been demonstrated with the PREDIMED Study. This randomized, controlled, multicenter clinical trial had three intervention groups: two with a traditional MedDiet supplemented with extra virgin olive oil and nuts, respectively, and low-fat diet control (6). In addition, a meta-analysis of 50 epidemiological and clinical trials (534,906 participants) determined that adherence to the MedDiet was associated with a reduced risk of metabolic syndrome (7).

Obesity is characterized by an increase of adipose tissue which, due to its involvement in metabolic regulation functions, has been acknowledged as an endocrine tissue organ. A maze of cytokines, hormones, substrates, and products, with both pro-and anti-inflammatory effects, regulate feelings of hunger and satiety through signals from the gastrointestinal tract and adipose tissue. Dietary interventions accompanied by weight loss have been shown in mid-and long-term programs to substantially influence satiety hormones. Feelings of hunger and satiety involve complex interactions between ghrelin and leptin in the hypothalamus which integrates both signals to regulate the body’s energy homeostasis (8–10). Leptin, an adipose tissue-specific adipokine, is crucial in the control of appetite, energy expenditure, behavior, and glucose metabolism. It crosses the blood–brain barrier and acts on specific receptors to decrease appetite and increase energy expenditure. Reduction in leptin levels has been observed short and mid-term (11, 12), while fewer studies have demonstrated MedDiet effectivity beyond a 12-month intervention (13). Physical activity and a caloric-restricted diet have jointly been reported to augment leptin decrease (14). Ghrelin, an endogenous peptide mainly secreted by the gut, contributes to orexigenic stimulus thus increasing appetite (15, 16). Higher circulating levels have been observed in short-term, with lesser evidence after 1 year of initial weight loss (13).

By interacting with different cell lineages (17–20), leptin acts as a pro-inflammatory adipokine and increases C-reactive protein levels in primary hepatocytes and human coronary endothelial cells (21, 22). Low-grade chronic inflammation is associated with adiposity, advanced age, dyslipidemia, and hyperglycemia. Inflammatory status can be counteracted by modifying diet patterns, including moderate physical activity (23–25). Several biomarkers engage in the complex process of inflammation, such as C-reactive protein, considered to reflect inflammatory reactions in atherosclerotic vessels, as well as circulating cytokines and necrosis in acute myocardial infarction (26). Plasminogen activator inhibitor-1 (PAI-1), a physiological inhibitor of plasminogen, acts as a biomarker of a pro-thrombotic state. MedDiet interventions have been reported to ameliorate pro-thrombotic status decreasing PAI-1 serum levels (27, 28). Smoking, alcohol consumption, and age are positively correlated with PAI-1 levels (29).

White adipose tissue has been broadly accepted as a metabolic active organ. However, some of its peptides are unclear, for instance, resistin, an antagonist polypeptide of insulin action that may play a role in obesity (30). Controversial results have been obtained regarding the identification of changes in its levels in both obesity and diabetes mellitus (31, 32). Regarding visfatin, an adipokine with arguably insulin-mimetic effects (33) and which is highly expressed in visceral fat (34, 35) appears to be upregulated in patients with obesity (36) and type 2 diabetes mellitus (37). Results, however, are inconsistent with respect to insulin sensitivity, waist circumference, body mass index (BMI), and HbA1c (38–40).

Our objective is to assess whether an intervention with a restrictive MedDiet plus physical activity promotion, versus a non-restrictive MedDiet, is associated with an improvement in satiety-related hormones, weight loss, pro-inflammatory biomarkers, and glucose/lipid metabolism at mid-and long-term (6-and 12-month follow-ups). In addition, we will establish the association of these markers with weight loss irrespective of the intervention group.

## Materials and methods

### Study design and population recruitment

The PREDIMED-PLUS is a multicenter lifestyle intervention with 6,874 eligible participants. It is a 6-year randomized trial conducted in 23 Spanish centers with a large cohort presenting metabolic syndrome recruited from primary healthcare centers. Inclusion criteria were: men aged 55–75 years and women 60–75 years, with overweight/obesity (BMI: 27–40), and meeting at least three metabolic syndrome components at baseline (41, 42).

Patients were randomly allocated either to the intervention group or control (41). Those in the former followed an energy-reduced MedDiet with physical activity promotion and behavioral support so as to meet specific weight loss objectives. The participants received recommendations based on a 17-item energy-restricted score. In addition, physical activity counseling to gradually increase exercise intensity to 150 min/week, and attitudinal lifestyle advice through frequent sessions with dietitians (both individual and collective), were provided. Participants in the control group received educational sessions on an *ad libitum* MedDiet based on a 14-item non-energy-restricted score. No specific advice for increasing physical activity or losing weight was provided.

Regarding the individual sessions, participants in both groups received periodical group sessions and telephone calls (once a month in the intensive intervention group and two times a year in the control one).

Adherence to diet was assessed with a previously validated 14-item questionnaire employed in the PREDIMED Study for the control group (43, 44), which was adapted to the 17-item energy-restricted diet questionnaire for the intervention group. According to the score obtained, the scale was estimated as approximate tertiles: low (≤ 7), medium (8–10), and high (11–17) (45). Physical activity practice was evaluated at the beginning of the study and during follow-up. Participants reported activities through the Regicor Short Physical Activity Questionnaire, a validated version adapted from the Minnesota leisure time physical activity questionnaire (46, 47). Physical activity was measured in MET⋅min/week.

Hormone and inflammation-related determinations were performed in a subsample of 300 patients at baseline, with measurements at 6-and 12-month follow-ups of 298 and 266 subjects, respectively. The sample size of glycosylated A1c hemoglobin (HbA1c) was made up of 300, 353, and 369 individuals at the three visits, respectively. Due to sample availability, high sensitivity C-reactive protein (hs-CRP) was analyzed in 228 individuals.

### Laboratory, anthropometric, and clinical data

The following information was gathered before and after the intervention: (i) the participants’ general clinical status (sex, age, BMI, waist circumference, systolic/diastolic blood pressure); (ii) adherence to the energy-reduced MedDiet (with a 17-point questionnaire); and (iii) levels of physical activity. Sample collection was performed after an overnight fasting period at baseline, 6-months, and 12-months of follow-up. Venous blood samples were collected in vacuum tubes with a silica clot activator and K_2_-EDTA anticoagulant (Becton Dickinson, Plymouth, United Kingdom) to yield serum and plasma, respectively. Serum tubes were centrifuged after the completion of the coagulation process, and plasma tubes immediately after collection, both for 15 min at 1.700 g room temperature. With the exception of HbA1c which was analyzed with K_2_-EDTA anticoagulated whole blood, the following analytes were quantified in serum with an ABX Pentra-400 auto-analyzer (Horiba-ABX, Montpellier, France): glucose, HbA1c, triglycerides, high-density lipoprotein (HDL) cholesterol, and total cholesterol. Low-density lipoprotein (LDL) cholesterol was calculated according to the Friedewald formula whenever triglycerides were < 300 mg/dL. Remnant-C was estimated as total cholesterol minus LDL cholesterol minus HDL cholesterol. Finally, leptin, ghrelin, glucagon-like peptide-1 (GLP-1), C-peptide, glucagon, insulin, PAI-1, resistin, and visfatin were simultaneously analyzed in plasma by Bio-Plex Pro methodology, a bead-based multiplexing technology with specific capture antibodies coupled with magnetic beads to discriminate analytes using an XMAG-Luminex assay (Bio-Rad, Hercules, CA, USA). The fluorescence signal was read on a Bio-Plex 200 equipment (Bio-Rad) (14). After several washes to remove unbound protein, a biotinylated detection antibody conjugated with fluorescent dye reporter. Homeostatic model assessment for insulin resistance (HOMA) was calculated as fasting plasma glucose (mg/dL) x fasting serum insulin (μ units/mL)/405. The inter-assay coefficients of variation (CVs) of these determinations were between 4.92 and 12.43%, except for GLP-1 (24.11%) and vifastin (32.42%). Values under the methodological limit of detection were reported with the limit of detection itself. Leptin measurements from six individuals were removed from the database due to analytical sampling error, and two hs-CRP values were considered outliers.

### Statistical analysis

The assessment of the normality distribution of the variables was performed based on normality probability plots and boxplots. Continuous variables were normally shaped, except for triglycerides which were normalized by Napierian logarithm, and median and interquartile ranges were displayed. Lifestyle categorical variables were compared between groups with the Chi-square test.

A descriptive statistic table stratified by intervention and control group was summarized including mean values (or median if non-normally shaped), and mean differences between 6-and 12-month intervals. In addition, multivariate linear regression models adjusted for sex, age, energy intake baseline value, and baseline value of the variable under study were fitted. Mean differences between groups were estimated and 95% confidence intervals were reported. To identify possible statistical differences across time, we performed the paired *t*-test among baseline, 6 months, and 12 months in each group (Mann–Whitney *U* test was carried out for non-normal variables).

Weight loss and waist circumference changes were stratified according to the tertiles of the population at the different time points (baseline, 6 months, and 12 months). To estimate the extent of variation among the first, second, and third tertiles, the analysis of variance was calculated by adjusting for baseline value and baseline weight. Major weight and waist circumference losses corresponded to the first tertile. The linear mixed-effect models were constructed considering potential significant covariates with age, sex, time, weight, and adherence to MedDiet as fixed effects. Given that time affects individuals differently, it was contemplated as a varying covariate and a random slope constructed. The model contains both linear and quadratic time components so as to determine which trend better fits the model. We also included possible interaction between sex and weight, using the latter to correct the model in all variables (except for weight itself). Linear mixed-effect estimation was carried out with the use of restricted maximum likelihood. Graphical representation of variables that showed significant results for the and/or group: time interaction (linear and/or quadratic component) was performed. In addition, analysis of 1-year weight loss correlation with these variables was calculated with Pearson’s correlation formula. A *p*-value of < 0.05 was considered significant.

#### Sample size

Accepting an alpha risk of 0.05 and a beta risk of < 0.2 in a bilateral contrast, 116 subjects in both groups allow the detection of a difference ≥ 1,2 pg/mL for leptin circulating levels, when the standard deviation is assumed to be 3.26 pg/mL.

## Results

Our study population was a sample of 407 (215 women) participants from the IMIM (Hospital del Mar Research Institute) site within the framework of the PREDIMED PLUS Study. The mean age was 65.44 years (± 4.62 years). With respect to participants’ lifestyles at baseline, the diet and physical activity questionnaire scores did not show significant differences between groups, and they met the minimal physical activity requirements suggested by the American Heart Association (450–750 MET⋅min⋅week^−1^) (48). Diabetes, dyslipidemia, hypertension, and smoking conditions were equally distributed between the two groups without significant differences.

Baseline, 6-and 12-month follow-ups, characteristics of continuous variables regarding clinical features, lifestyle, lipid/glucose metabolism, satiety-related hormones, and studied pro-inflammatory markers are shown in Table 1. The main food items and nutritional parameters are shown in Table 2. In comparison to the control group, the adjusted multivariate of MedDiet adherence, physical activity, weight, waist circumference, remnant cholesterol, triglyceride levels, and HDL cholesterol showed an improvement at 6-month follow-up which was maintained at 12 months. Systolic and diastolic blood pressure presented significant improvements at 6-month follow-up but did not reach significance at 12 months. Regarding carbohydrate metabolism, we found differences between the two groups at 6-and 12-month follow-ups in HOMA, insulin, and C-peptide. Borderline inter-group *P*-value these explanations were aimed to clarify the meaning of borderline to reviewer 2. Borderline inter-group was observed for glucose at 6 and 12 months [a tendency to ameliorate results over time: β_6*m*_ = −3.58 (−7.39, 0.23) and β_12*m*_ = −4.22 (−9.16, 0.72)] and a significant decrease for HbA1c only at 12 months. Changes in leptin and PAI-1 levels were reported at 12 months, with a 6-month *P*-value close to significance in the case of PAI-1. Mean multivariate-adjusted differences (95% CI) for 6-and 12-month follow-ups were estimated and are depicted in common units of baseline standard deviations in Supplementary Figure 1.

**TABLE 1:** Baseline and 6-and 12-month changes (mean and standard deviation) stratified in the control and intervention groups of the participants on the 17-item questionnaire, physical activity, biomarkers, and anthropometric measurements, lipid profile, carbohydrate metabolism, and hormones. Adjusted for sex and age.

	Control group			Intervention group			Control group vs. Intervention group		
	Baseline	6 month-change	12 month-change	Baseline	6 month-change	12 month-change	Baseline	6 month-adjusted model	12 month-adjusted model
**Diet adherence and physical activity**									
Mediterranean diet adherence (17-point item score)	7.18 (2.36)	3.03* (3.03)	2.55 + ^ (2.94)	7.52 (2.60)	4.13* (3.27)	3.89^ (3.38)	0.30 (−0.17, 0.77)	1.37 (0.88, 1.86)	1.62 (1.13, 2.12)
Physical activity (MET⋅min/week)	2477 (2132.37)	275.77 (2311.75)	367.91^ (2272.46)	2648 (2216.67)	838.78* (2430.42)	872.23 ^ (2207.52)	150.64 (−269.65, 570.93)	649.28 (233.83, 1064.72)	591.67 (206.63, 976.71)
**Lipid profile**									
Total cholesterol (mg/dL)	218.30 (41.22)	−3.69 (31.71)	−0.16 (33.93)	221.88 (41.65)	−4.50* (32.54)	−1.70 (32.66)	3.62 (−4.06, 11.30)	0.69 (−4.95, 6.34)	−0.34 (−6.53, 5.85)
HDL cholesterol (mg/dL)	54.29 (11.06)	0.91 (6.89)	−0.13 + (7.06)	52.73 (11.21)	2.53* (7.79)	2.62 ^ (7.83)	−1.74 (−3.69, 0.22)	1.35 (−0.10, 2.79)	2.29 (0.84, 3.74)
LDL cholesterol (mg/dL)	133.67 (35.19)	−4.17* (25.22)	2.03 + (28.77)	139.22 (38.03)	−3.03 (27.71)	−0.36 (27.70)	5.67 (−1.45, 12.80)	3.44 (−1.38, 8.26)	−0.41 (−5.74, 4.92)
Triglycerides (mg/dL)	144 [107: 186]	−5.93 (55.03)	−13.48^ (55.65)	134 [104:182]	−13.48* [−38:6]	−16^ [−42:10]	0.00 (−0.09, 0.08)	−0.12 (−0.18, −0.07)	−0.08 (−0.14, −0.02)
Remnant cholesterol (mg/dL)	29.13 (10.60)	−1.08 (8.66)	−1.52^ (8.05)	28.52 (10.88)	−3.72* (7.82)	−3.03^ (9.49)	−0.48 (−2.62, 1.67)	−2.81 (−4.30, −1.33)	−1.78 (−3.43, −0.13)
**Blood pressure and anthropometric measurements**									
Systolic pressure (mmHg)	139.25 (13.46)	−2.04* (14.30)	−3.64^ (14.61)	140.01 (12.90)	−6.38* (13.90)	−5.18^ (15.43)	0.63 (−1.92, 3.18)	−3.90 (−6.46, −1.35)	−1.09 (−3.79, 1.61)
Diastolic pressure (mmHg)	74.59 (10.74)	−0.81 (11.22)	−2.10^ (11.11)	75.59 (9.77)	−4.99* (10.20)	−4.06^ (10.87)	1.13 (−0.80, 3.05)	−3.39 (−5.24, −1.53)	−1.21 (−3.13, 0.71)
Weight (kg)	88.98 (13.71)	−2.66* (3.47)	−2.67^ (3.99)	87.54 (13.87)	−6.31* (4.09)	−7.41 + ^ (4.07)	−1.12 (−3.46, 1.21)	−3.74 (−4.46, −3.03)	−4.84 (−5.60, −4.08)
Waist circumference (cm)	111.47 (9.56)	−2.81* (3.70)	−2.83 ^ (4.48)	110.25 (9.74)	−6.21* (4.73)	−7.28 + ^ (4.37)	−1.10 (−2.84, 0.64)	−3.52 (−4.33, −2.70)	−4.57 (−5.44, −3.70)
**Carbohydrate metabolism**									
HOMA	3.14 (3.18)	−0.30* (1.82)	−0.34 ^ (1.52)	2.97 (2.17)	−0.75* (1.24)	−0.70^ (1.38)	−0.17 (−0.79, 0.45)	−0.49 (−0.83, −0.14)	−0.38 (−0.70, −0.06)
Glucose (mg/dL)	119.90 (33.19)	−2.92 (22.11)	−0.53 (27.27)	120.86 (31.32)	−6.67* (17.69)	−4.95^ (21.75)	1.04 (−5.25, 7.33)	−3.58 (−7.39, 0.23)	−4.22 (−9.16, 0.72)
HbA1c (%)	6.40 (1.11)	−0.27* (0.72)	−0.06 + (0.55)	6.33 (0.84)	−0.33* (0.45)	−0.21 + ^ (0.50)	−0.07 (−0.30, 0.15)	−0.06 (−0.19, 0.07)	−0.14 (−0.27, −0.02)
Insulin (pg/mL)	351.81 (230.53)	−24.19 (160.44)	−33.22^ (142.44)	336.79 (189.70)	−77.56* (129.63)	−79.59^ (131.15)	−14.65 (−62.49, 33.19)	−57.09 (−90.06, −24.12)	−49.39 (−79.44, −19.33)
Glucagon (pg/mL)	455.03 (163.32)	−36.43* (114.51)	−39.24^ (115.95)	446.44 (154.60)	−47.00* (128.95)	−48.99^ (125.23)	−6.35 (−41.12, 28.43)	−13.46 (−39.90, 12.98)	−11.33 (−38.03, 15.37)
C-peptide (pg/mL)	1054.99 (522.52)	−68.82* (303.26)	−89.74^ (313.09)	1066.02 (432.07)	−155.66* (327.24)	−170.98^ (316.98)	11.15 (−97.34, 119.65)	−83.93 (−149.45, −18.41)	−82.90 (−149.68, −16.12)
GLP_1 (pg/mL)	172.39 (134.29)	−8.33 (81.05)	−12.46 (98.24)	166.78 (120.69)	−9.71 (91.22)	−23.20^ (82.12)	−5.75 (−34.43, 22.93)	−4.45 (−22.92, 14.01)	−13.74 −33.43, 5.95)
**Hormones and inflammation biomarkers**									
Ghrelin (pg/mL)	807.38 (415.02)	−30.86 (245.98)	−31.21 (236.83)	831.31 (412.41)	−11.05 (207.24)	5.69 (227.47)	26.33 (−67.05, 119.72)	26.96 (−24.03, 77.95)	40.68 (−12.90, 94.25)
Leptin (pg/mL)	7746.99 (4152.80)	−730.11 (2486.83)	−776.03 (2727.56)	7246.06 (3850.42)	−1020.43 (3141.41)	−1310.85 (2524.41)	−385.77 (−1145.57, 374.04)	−425.74 (−1036.51, 185.04)	−698.56 (−1295.48, −101.64)
PAI_1 (pg/mL)	2631.62 (838.39)	−250.14* (715.26)	−129.02 + ^ (729.33)	2652.39 (828.08)	−425.63* (837.60)	−371.54^ (692.88)	29.66 (−158.92, 218.24)	−153.42 (−312.00, 5.16)	−252.41 (−403.90, −100.92)
Resistin (pg/mL)	4625.87 (2138.00)	−286.75* (1670.16)	−362.79^ (1815.51)	4254.35 (1635.32)	−74.65* (1343.08)	−12.26 + ^ (1176.19)	−378.91 (−812.86, 55.05)	55.85 (−247.20, 358.89)	151.36 (−160.26, 462.98)
Visfatin (pg/mL)	1309.09 (1620.44)	−302.97* (1314.76)	−300.98^ (1375.44)	1194.63 (1093.55)	−270.33* (668.66)	−258.90^ (613.18)	−93.64 (−387.15, 199.87)	−52.22 (−205.86, 101.41)	−47.97 (−214.70, 118.76)
hs-PCR (mg/dL)	0.45 (0.61)	−0.07 (0.81)	−0.13^ (0.47)	0.45 (0.61)	−0.13* (0.63)	−0.11 (0.81)	0.00 (−0.15, 0.16)	−0.01 (−0.09, 0.08)	0.02 (−0.11, 0.15)

*#*Median and interquartile range were displayed in non-normal distributed variables *: significant *P-value* between baseline and 6-month follow-up; + : significant *P-value* between 6-month follow-up and 12-month follow-up; ^: significant *P-value* between baseline and 12-month follow-up.

**TABLE 2:** Baseline and differences at 6-and 12-month follow-ups (mean and standard deviation) stratified in the control and intervention groups in the consumption of key food items and dietary parameters between the control and intensive group adjusted for the baseline value.

	Baseline	6 month-change	12 month-change	Baseline	6 month-change	12 month-change	Baseline	6 month-adjusted model	12 month-adjusted model
Energy intake (kcal/day)	2464.42 (548.70)	−135.32 (559.65)	−160.20 (576.76)	2357.21 (528.53)	−111.46 (585.36)	−110.20 (581.21)	< 0.05	0.054	0.368
Carbohydrates (g/day)	227.24 (66.12)	−20.31 (73.14)	−20.84 (64.67)	218.22 (69.56)	−22.88 (72.54)	−18.93 (75.03)	0.181	< 0.05	0.241
Protein (g/day)	105.80 (19.94)	2.07 (20.69)	−1.74 (22.29)	102.25 (19.77)	6.84 (23.13)	6.23 (22.15)	0.072	0.288	< 0.05
Total fat (g/day)	118.22 (28.89)	−4.14 (30.72)	−5.15 (33.56)	113.22 (28.08)	−2.12 (33.58)	−3.45 (33.79)	0.077	0.272	0.255
Saturated fatty acids (g/day)	30.80 (9.50)	−5.47 (9.11)	−5.84 (9.62)	29.25 (9.03)	−6.18 (9.58)	−6.19 (9.89)	0.093	< 0.001	< 0.05
Monounsaturated fatty acids (g/day)	61.05 (15.04)	1.83 (19.54)	2.11 (20.07)	58.08 (14.90)	5.59 (21.29)	4.85 (19.85)	< 0.05	0.451	0.968
Polyunsaturated fatty acids (g/day)	19.01 (5.83)	3.12 (6.90)	2.29 (7.32)	18.82 (6.84)	3.64 (7.78)	2.62 (8.19)	0.761	0.528	0.800
Cholesterol (mg/day)	426.16 (105.26)	−40.19 (106.38)	−48.23 (121.61)	418.58 (118.16)	−35.06 (124.82)	−41.95 (132.46)	0.495	0.934	0.733
Trans-fatty acids (g/day)	0.72 (0.41)	−0.27 (0.39)	−0.28 (0.43)	0.70 (0.44)	−0.37 (0.45)	−0.37 (0.46)	0.579	< 0.001	< 0.001
Linolenic acid	1.74 (0.67)	0.52 (0.84)	0.37 (0.95)	1.72 (0.78)	0.63 (0.98)	0.44 (0.92)	0.775	0.248	0.538
Carbohydrate percentage (%)	36.72 (5.44)	−1.34 (6.41)	−1.01 (5.90)	36.72 (5.89)	−2.06 (6.67)	−1.35 (6.71)	1	0.076	0.462
Protein percentage (%)	17.44 (2.46)	1.28 (2.98)	0.84 (2.79)	17.62 (2.68)	1.88 (3.02)	1.83 (3.27)	0.481	< 0.001	< 0.001
Total fat percentage (%)	43.22 (5.36)	0.90 (6.52)	0.97 (6.14)	43.33 (5.70)	1.27 (6.70)	0.64 (6.93)	0.837	0.203	0.526
Saturated fatty acid percentage (%)	11.18 (1.97)	−1.44 (2.16)	−1.48 (1.96)	11.09 (1.82)	−1.86 (1.95)	−1.91 (1.99)	0.635	< 0.05	< 0.001
Monounsaturated fatty acid percentage (%)	22.41 (3.58)	1.91 (5.33)	2.27 (4.86)	22.34 (4.10)	3.16 (5.55)	2.89 (5.49)	0.856	0.004	0.219
Polyunsaturated fatty acid percentage (%)	6.95 (1.65)	1.64 (2.25)	1.37 (2.15)	7.21 (2.17)	1.82 (2.48)	1.38 (2.51)	0.185	0.014	0.198
Meat and meat products (g/day)	174.48 (59.31)	−17.35 (60.32)	−24.14 (61.51)	166.54 (52.82)	−7.08 (60.80)	−9.02 (63.08)	0.155	0.279	< 0.05
Fish (g/day)	120.91 (42.98)	17.11 (49.01)	6.91 (47.91)	126.49 (46.18)	15.53 (60.00)	14.27 (57.86)	0.207	0.523	0.005
Vegetables (g/day)	343.34 (149.73)	42.53 (186.18)	33.25 (171.62)	354.26 (122.21)	49.97 (176.83)	57.45 (143.06)	0.422	0.226	< 0.05
Total cereals (g/day)	129.44 (58.75)	−5.29 (69.71)	−7.00 (61.00)	119.38 (63.50)	−0.15 (63.61)	6.69 (78.60)	0.098	0.248	0.450
Dairy products (g/day)	370.65 (181.52)	14.88 (209.43)	−10.69 (208.67)	339.42 (168.83)	27.83 (214.99)	35.79 (189.12)	0.073	0.629	0.105
Nuts (g/day)	15.69 (15.92)	21.24 (26.01)	19.97 (25.91)	15.83 (16.73)	28.66 (25.76)	25.12 (25.57)	0.933	< 0.05	< 0.05
Fruit (g/day)	351.48 (174.25)	0.63 (224.44)	35.68 (223.37)	351.14 (174.26)	19.97 (221.11)	22.88 (209.81)	0.984	0.255	0.479
Legumes (g/day)	20.53 (10.15)	3.99 (12.26)	3.35 (13.21)	19.73 (9.02)	7.26 (12.01)	5.32 (11.78)	0.399	0.007	0.145
Olive oil (g/day)	47.77 (13.78)	−0.11 (17.34)	1.35 (16.49)	45.47 (14.44)	1.89 (17.33)	2.17 (16.41)	0.100	0.906	0.244
Virgin olive oil (g/day)	30.39 (20.44)	13.40 (22.33)	13.94 (21.70)	31.61 (20.07)	12.29 (21.66)	13.61 (21.43)	0.544	0.963	0.580
Sunflower oil (g/day)	0.74 (2.81)	−0.65 (2.77)	−0.40 (2.56)	1.35 (6.44)	−1.31 (6.65)	−1.19 (6.24)	0.214	0.755	0.327
Dietary fiber (g/day)	24.93 (7.18)	5.80 (8.72)	5.08 (8.75)	25.25 (6.93)	7.29 (8.83)	6.93 (8.16)	0.645	< 0.05	< 0.001
Alcohol (g/day)	9.76 (12.52)	−3.59 (10.38)	−3.36 (8.86)	8.05 (9.84)	−4.02 (9.45)	−4.04 (7.81)	0.128	< 0.05	< 0.05

As expected, the weight loss tertiles showed improvements at mid-and long-term follow-up for MedDiet adherence and physical activity practice regardless of the group. In particular, we observed changes in the triglyceride-related measurements (total cholesterol, HDL cholesterol, triglycerides, and remnant cholesterol), systolic/diastolic blood pressure, and carbohydrate metabolism (HOMA, HbA1c, insulin, glucagon, C-peptide, GLP-1). In addition, changes in leptin, PAI-1, and visfatin levels were observed at 6-and 12-month follow-ups (Table 3). Waist circumference change tertiles showed similar results to body weight tertiles (Table 4).

**TABLE 3:** Tertiles of weight loss change (mean, standard deviation, and their comparison) adjusted for weight and baseline value of the participants on the 17-item questionnaire, physical activity, biomarkers and anthropometric measurements, lipid profile, carbohydrate metabolism, and hormones.

	First tertile of 6-month weight-loss change	Second tertile of 6-month weight-loss change	Third tertile of 6-month weight-loss change	Global *P-value*	First tertile of 12-month weight-loss change	Second tertile of 12-month weight-loss change	Third tertile of 12-month weight-loss change	Global *P-value*
**Diet adherence and physical activity**								
Mediterranean diet adherence (17-point item score)	4.93 (3.12)	3.31* (2.91)	2.42+ ^ (3.04)	< 0.001	4.56 (3.10)	2.70* (3.18)	2.34 + ^ (2.98)	< 0.001
Physical activity (MET⋅min/week)	1084.68 (2503.52)	390.80* (2197.49)	167.02^ (2356.66)	< 0.001	933.72 (2398.89)	596.32* (1969.18)	311.94^ (2346.88)	< 0.05
**Lipid profile**								
Total cholesterol (mg/dL)	−8.99 (30.57)	−4.73 (36.49)	1.64^ (28.05)	< 0.05	−2.52 (32.39)	−3.51 (36.13)	3.76 (30.54)	0.414
HDL cholesterol (mg/dL)	3.34 (8.04)	0.71* (6.96)	0.96^ (6.80)	< 0.05	3.54* (7.87)	0.71* (7.12)	−0.73^ (7.11)	< 0.001
LDL cholesterol (mg/dL)	−8.00 (25.82)	−1.21 (30.34)	−1.29^ (22.28)	0.113	−1.17 (27.43)	0.03 (30.99)	4.05 (25.70)	0.370
Triglycerides (mg/dL)	−33.70 (61.93)	−14.25* (53.21)	1.22^ (53.76)	< 0.001	−27.33 (53.10)	−21.30* (57.28)	−2.96 + ^ (60.50)	< 0.001
Remnant cholesterol (mg/dL)	−5.35 (8.44)	−1.90* (7.54)	0.33^ (8.05)	< 0.001	−4.72 (9.12)	−2.59* (8.30)	0.90 + ^ (8.12)	< 0.001
**Blood pressure and anthropometric measurements**								
Systolic pressure (mmHg)	−7.71 (13.62)	−4.48* (14.46)	−0.14 + ^ (13.74)	< 0.05	−7.86 (13.49)	−3.96* (15.40)	−1.18^ (15.50)	< 0.05
Diastolic pressure (mmHg)	−5.41 (10.11)	−2.84 (11.21)	−0.21 + ^ (10.89)	< 0.001	−4.71 (10.98)	−3.39 (11.29)	−0.95 + ^ (10.51)	< 0.05
Waist circumference (cm)	−8.20 (4.24)	−3.88* (3.28)	−1.19 + ^ (2.87)	< 0.001	−9.32 (4.64)	−4.45* (2.83)	−1.05 + ^ (3.21)	< 0.001
**Carbohydrate metabolism**								
HOMA	−0.94 (1.25)	−0.38* (2.08)	−0.02^ (1.01)	< 0.001	−1.02 (1.18)	−0.49* (1.61)	0.13 + ^ (1.36)	< 0.001
Glucose (mg/dL)	−10.73 (19.68)	−4.47* (21.84)	1.20 + ^ (16.96)	< 0.001	−8.16 (18.06)	−2.87 (31.30)	3.46^ (21.29)	< 0.001
HbA1c (%)	−0.44 (0.54)	−0.30 (0.77)	−0.07 + ^ (0.33)	< 0.001	−0.35 (0.47)	−0.05* (0.48)	0.06^ (0.57)	< 0.05
Insulin (pg/mL)	−93.90 (134.85)	−30.79* (177.62)	−5.45^ (102.70)	< 0.001	−108.89 (124.15)	−43.90* (143.14)	1.47^ (126.86)	< 0.001
Glucagon (pg/mL)	−72.75 (131.53)	−29.64* (106.92)	−7.82^ (112.95)	< 0.001	−83.56 (126.80)	−30.87* (102.37)	−5.49^ (116.94)	< 0.001
C-peptide (pg/mL)	−207.60 (322.66)	−71.29* (299.50)	−9.15^ (292.38)	< 0.001	−227.06 (305.38)	−104.96* (303.46)	−26.49^ (313.63)	< 0.001
GLP_1 (pg/mL)	−14.42 (84.28)	−3.39* (99.23)	−7.89 (68.07)	0.105	−21.13 (75.58)	−18.21 (88.02)	−12.87^ (111.02)	0.109
**Hormones and inflammation biomarkers**								
Ghrelin pg/mL)	−32.28 (205.27)	−21.59 (195.07)	−3.61 (295.65)	0.653	−8.91 (225.22)	−20.47 (224.18)	−9.35 (253.85)	0.966
Leptin (pg/mL)	−1549.05 (3235.36)	−724.65* (2355.01)	22.19^ (2400.70)	< 0.001	−1789.81 (2299.54)	−1194.54* (2562.08)	156.79 + ^ (2762.06)	< 0.001
PAI_1 pg/mL)	−484.66 (811.69)	−261.77* (786.25)	−193.61^ (684.39)	< 0.05	−464.06 (640.85)	−239.00* (718.15)	31.85 + ^ (735.85)	< 0.001
Resistin (pg/mL)	−142.90 (1542.54)	−183.42 (1322.29)	-−253.10 (1745.68)	0.588	−106.24 (1215.95)	−129.09 (1457.42)	−369.27 (1971.13)	0.710
Visfatin (pg/mL)	−352.27 (702.77)	−227.70 (568.43)	−263.99^ (1780.08)	< 0.001	−388.93 (627.74)	−226.48* (553.56)	−192.99^ (1765.19)	< 0.001
hs-PCR (mg/dL)	−0.21 (0.72)	−0.42 (2.94)	−0.04 (0.33)	0.297	−0.23 (0.65)	−0.01 (0.86)	−0.54 (3.35)	0.141

*: significant *P-value* between first and second tertile; + : significant *P-value* between second and third tertile; ^: significant *P-value* between first and third tertile.

**TABLE 4:** Tertiles of waist circumference change (mean, standard deviation, and their comparison) adjusted for weight and baseline value of the participants on the 17-item questionnaire, physical activity, biomarkers and anthropometric measurements, lipid profile, carbohydrate metabolism, and hormones.

	First tertile of 6-month waist circumference change	Second tertile of 6-month waist circumference change	Third tertile of 6-month waist circumference change	Global *P-value*	First tertile of 1-year waist circumference change	Second tertile of 1-year waist circumference change	Third tertile of 1-year waist circumference change	Global *P-value*
**Diet adherence and physical activity**								
Mediterranean diet adherence (17-point item score)	4.76 (3.12)	3.38* (3.04)	2.55 + ^ (3.04)	< 0.001	4.73 (3.34)	2.92* (2.58)	2.48 + ^ (2.87)	< 0.001
Physical activity (MET⋅min/week)	932.61 (2453.74)	617.23 (2262.68)	122.75 + ^ (2462.50)	< 0.05	1202.29 (2242.48)	531.83* (2238.96)	263.94 + ^ (2051.53)	< 0.001
**Lipid profile**								
Total cholesterol (mg/dL)	−5.33 (31.52)	−4.50 (33.51)	−2.09 (31.08)	0.796	−3.66 (30.10)	2.11 (34.04)	−1.36 (35.69)	0.168
HDL cholesterol (mg/dL)	2.89 (8.22)	1.63 (6.61)	0.36^ (7.05)	< 0.05	2.01 (7.33)	2.60 (7.65)	−1.19 + ^ (7.24)	< 0.001
LDL cholesterol (mg/dL)	−3.70 (27.74)	−2.60 (26.89)	−4.74 (24.42)	0.567	0.74 (25.35)	1.77 (29.73)	−0.13 (29.73)	0.231
Triglycerides (mg/dL)	−31.64 (64.98)	−17.41* (53.28)	5.09 + ^ (48.90)	< 0.001	−33.86 (54.90)	−12.75* (50.83)	−5.30^ (64.12)	< 0.001
Remnant cholesterol (mg/dL)	−4.41 (8.71)	−2.81* (7.26)	0.60 + ^ (8.39)	< 0.001	−5.65 (8.85)	−1.48* (7.86)	0.61 + ^ (8.65)	< 0.001
**Blood pressure and anthropometric measurements**								
Systolic pressure (mmHg)	−7.86 (14.34)	−4.32* (13.46)	0.51 + ^ (13.90)	< 0.001	−7.00 (13.94)	−4.02 (15.69)	−1.99^ (15.07)	< 0.05
Diastolic pressure (mmHg)	−5.02 (10.19)	−3.57 (10.68)	0.68 + ^ (11.31)	< 0.001	−3.72 (11.06)	−4.64 (10.58)	−0.56 + (11.14)	< 0.05
Weight (kg)	−8.00 (4.49)	−4.07* (1.97)	−0.81 + ^ (2.24)	< 0.001	−9.16 (4.60)	−4.83* (2.59)	−1.15 + ^ (2.58)	< 0.001
**Carbohydrate metabolism**								
HOMA	−0.83 (1.30)	−0.68 (1.54)	0.12 + ^ (1.78)	< 0.001	−0.95 (1.22)	−0.57* (1.29)	−0.05 + ^ (1.71)	< 0.001
Glucose (mg/dL)	−9.30 (19.90)	−5.65 (20.32)	1.84 + ^ (18.58)	< 0.001	−5.92 (28.74)	−2.98 (20.17)	1.12^ (24.32)	0.051
HbA1c (%)	−0.45 (0.51)	−0.30* (0.71)	−0.09 + ^ (0.49)	< 0.001	−0.36 (0.49)	−0.14* (0.43)	0.10 + ^ (0.58)	< 0.001
Insulin (pg/mL)	−82.04 (132.49)	−59.17* (130.24)	5.55 + ^ (176.87)	< 0.001	−95.44 (123.25)	−66.84* (128.61)	−5.78 + ^ (149.81)	< 0.001
Glucagon (pg/mL)	−78.51 (122.47)	−31.70* (118.85)	−6.87^ (112.42)	< 0.001	−69.39 (139.30)	−47.78* (96.20)	−14.95 + ^ (120.76)	< 0.001
C-peptide (pg/mL)	−184.51 (354.04)	−105.02* (277.13)	−21.46^ (299.70)	< 0.001	−216.09 (296.89)	−139.44* (295.42)	−34.91 + ^ (336.88)	< 0.001
GLP_1 (pg/mL)	−7.86 (86.89)	−11.69 (93.79)	−6.64 (73.08)	0.620	−12.84 (73.14)	−25.92 (88.71)	−13.53 (107.03)	0.239
**Hormones and inflammation biomarkers**								
Ghrelin (pg/mL)	−36.13 (212.62)	−4.74 (183.25)	−25.43 (296.16)	0.657	−23.74 (259.87)	8.61 (182.88)	−26.26 (254.03)	0.541
Leptin (pg/mL)	−1654.22 (3115.84)	−545.42* (2525.77)	−281.55^ (2583.37)	< 0.001	−1741.04 (2490.24)	−924.76* (2237.87)	−468.22^ (3030.65)	< 0.001
PAI_1 (pg/mL)	−561.69 (620.62)	−295.04* (949.65)	−92.44^ (609.46)	< 0.001	−456.89 (603.42)	−243.57* (710.70)	−52.24 + ^ (785.81)	< 0.001
Resistin (pg/mL)	−166.84 (1586.23)	−137.04 (1494.89)	−275.84 (1492.57)	0.668	−225.48 (1522.45)	−40.72 (1367.82)	−317.87 (1727.57)	0.935
Visfatin (pg/mL)	−379.89 (695.73)	−308.23* (1494.60)	−136.14 ^ (571.59)	< 0.001	−355.01 (579.81)	−375.96* (1571.81)	−96.18^ (599.17)	< 0.001
hs-PCR (mg/dL)	−0.22 (0.77)	−0.39* (2.77)	−0.06^ (0.42)	< 0.05	−0.13 (0.87)	−0.17 (0.62)	−0.43 (3.01)	0.413

*: significant *P-value* between first and second tertile; + : significant *P-value* between second and third tertile; ^: significant *P-value* between first and third tertile.

Changes were graphically examined through linear mixed-effect models of cardiovascular risk factors at 6-and 12-month follow-ups to observe the behavior of the repeated measures in both groups. The time:group (linear and quadratic) interaction as a potential predictor of the outcome variable was significant in weight, waist circumference, HDL, and remnant cholesterol, systolic/diastolic blood pressure, triglycerides, and PAI-1 levels (Supplementary Figure 2). Pearson’s correlation at 1 year yielded a moderately positive correlation (*r* > 0.20) between weight loss and reduction of leptin, glucagon, PAI-1, HbA1c, and insulin levels. Comparably, moderately positive correlations (*r* > 0.20) between waist circumference changes and reduction of leptin, PAI-1, and insulin levels were observed. Weight change with moderate positive correlation was reported (Supplementary Figure 3).

## Discussion

The intervention with an energy-reduced MedDiet and physical activity, versus a non-reduced one, was associated with an improvement in weight, waist circumference, glucose metabolism, triglyceride-related lipid profile, satiety-related hormones (leptin), and pro-inflammatory markers (PAI-1) at mid-and long-term in subjects with metabolic syndrome.

Such changes being maintained over time have been previously reported. Moreover, it has been hypothesized that MedDiet pattern interventions lead to greater compliance and adherence rates, in fact, the number of dropouts registered in trials has been reported to be larger in the control groups (7, 49–52). The MedDiet fat component is of vegetable origin (olive oil and nuts) and includes an abundance of plant foods (vegetables, fruit, whole grains, and legumes), limited fish consumption, and red wine in moderation (usually during meals). The intake of red and processed meats, refined grains, potatoes, dairy products, and ultra-processed foods (ice cream, sweets, creamy desserts, industrial confectionery, and sugar-sweetened beverages) (41, 53).

The hypothesis that the MedDiet is an eating pattern that can be maintained in mid-and long-term with a high degree of acceptance has been reflected in several studies introducing behavioral and nutritional patterns into small population groups (52, 54, 55). During other interventions, several participants reported freshness and palatability of food, with variance across the studies regarding taste (56–58). Meal plans resulted in hedonic appreciation and satisfaction by most participants (58), although this differed according to age and dishes (57). There were, however, a number of barriers, such as dislike of some foods (including olive oil) and/or reduction of red meat. In addition to diet acceptability, various limitations have been reported such as the perception of expense, expectation of time commitment, perceived impact on body weight, and cultural differences (56, 58–60). Among a group of schoolchildren, a study found that food neophobia correlated negatively with certain healthy dietary habits, such as fruit and vegetable consumption.

The intervention group was based on a hypocaloric diet with moderate fat consumption of vegetable origin: olive oil, tree nuts, and peanuts. Furthermore, it was designed to augment complex carbohydrates and fiber-rich products. Moderate intake of monounsaturated fat in the form of olive oil is one of the cornerstones of MedDiet due to its culinary versatility. Its beneficial effects on the reduction of cardiovascular disease include cardioprotective characteristics, improvement in lipid profile (decrease in total and LDL cholesterol and an increase of HDL cholesterol) and blood pressure decrease, amelioration of LDL cholesterol oxidation and low-chronic inflammation, and anti-atherogenic properties (61–67).

### Weight and waist circumference

While short-term changes are relatively easy to accomplish, successfully maintaining them over time is considerably more difficult. The combination of diet-induced weight loss with exercise training has demonstrated greater improvement in cardiovascular risk factors than diet alone (68, 69). Our findings from the intervention group showed a decrease in waist circumference and weight at both 6-and 12-month follow-ups, and the comparison with the control was significant for both periods. The weight loss experienced by the control group, despite following a non-reduced diet, can be explained by their motivation to participate in a clinical trial for subjects with overweight/obesity. In the intervention group, the maximum weight loss was at 1 year. Such a finding is particularly relevant since in most studies on the effects of restrictive diets this occurs at 6 months followed by a reward effect. Interventions with hypocaloric diets which can be sustainable over time could, therefore, provide a better approach to weight loss. In this regard, a MedDiet is appropriate as its better palatability, due to its mainly vegetal content and use of olive oil leads to greater adherence.

### Leptin–Ghrelin binomial

Hyperleptinemia is a characteristic manifestation of obesity in humans. Resistance to leptin action in obesity has been suggested, and elevated circulating concentrations may be necessary to maintain sensitivity to hormone and energy homeostasis (70, 71). Leptin, as a polypeptide secreted by adipocytes, might be decreased as a result of fat mass reduction (72, 73). We observed a significant reduction in its levels after both the intervention and control groups. The former displayed an overall stronger decrease probably caused by the further reduction of anthropometric measurements. In fact, a significant reduction was reported comparing the intervention arm to the control at 12-month follow-up.

Individuals with overweight/obesity have typically lower circulating ghrelin levels. This adipogenic hormone seems to indicate downregulation in human obesity, supposedly as an adaptive mechanism in response to positive energy balance (74, 75). Diet-induced effects usually show an increase in circulating levels, although reversion to baseline levels at 12 months after a 6-month peak has been reported (76). Our cohort reflected an initial reduction followed by a minor increase in circulating levels in the intensive group, with no statistical significance.

### Carbohydrate metabolism-related hormones

Weight loss interventions lead to changes in carbohydrate homeostasis, and increased insulin sensitivity has been observed following dietary interventions, physical activity, and bariatric surgery (77, 78). Nevertheless, in contrast to isolated interventions, the combined effects of a restricted diet and physical exercise have been reported to improve to a greater extent such sensitivity and variables related to the cardiometabolic syndrome. In our intervention group, insulin levels decreased during the first 6 months and were maintained up to the 12-month follow-up. The control group also experienced a steady reduction although it presented higher levels at 6-and 12-month follow-ups. HOMA, C-peptide, HbA1c, and glucose levels followed a similar pattern.

Glucagon improvement caused by diet and exercise training has been reported in the literature. A meta-analysis made up of 29 interventions assessed body weight change, glucagon, insulin, and glucose fasting concentrations after two different weight reduction methods (bariatric surgery versus low-caloric diet intervention). More than half the diet interventions resulted in a decrease from 17 to 27%. The mean decrease in fasting glucagon, however, was not significantly different between both weight reduction approaches (77). Although no inter-group differences in the present study were obtained, a linear time component proved to be a predictor of weight loss regardless of the intervention.

### Lipid profile

Triglyceride reduction is crucial in the management of dyslipidemia, particularly atherogenic dyslipidemia which is highly prevalent in metabolic syndrome subjects. Atherogenic dyslipidemia is characterized by high circulating triglyceride levels and low levels of HDL cholesterol, and even optimal concentrations of LDL cholesterol. We have recently reported in subjects with overweight/obesity at high cardiovascular risk, that triglycerides and remnant cholesterol levels, but not LDL cholesterol, were associated with cardiovascular outcomes irrespective of other risk factors (79, 80). Triglyceride concentration is an independent risk factor for cardiovascular disease and is strongly associated with subcutaneous abdominal adipose tissue. In fact, it has been suggested that triglycerides could be a predictor of cardiovascular disease (79). The MedDiet has been previously studied as a dietary tool to improve metabolic syndrome and subsequent events (6, 79, 81). In this respect, our results show an overall triglyceride reduction in both groups, with a greater reduction in the intervention group than in the control. In concordance, we have recently reported that an energy-reduced MedDiet plus physical activity improves HDL-related triglyceride metabolism versus a non-reduced MedDiet without physical activity (82). Regarding remnant cholesterol, its levels follow a similar pattern to that of triglycerides. Although we did not observe changes after the intervention in total cholesterol, remnant cholesterol decreased in mid-and long-term versus the control group. Such a finding could be a good indicator that the intensive intervention shifted toward protection against cardiovascular risk.

High-density lipoprotein (HDL) cholesterol lipoproteins are known for their atheroprotective effects through a number of anti-inflammatory, anti-oxidative, anti-thrombotic, and anti-apoptotic properties (83, 84). An inverse association between triglycerides and HDL cholesterol concentrations usually occurs. In fact, HDL lipoproteins are catabolized faster in the presence of hypertriglyceridemia in non-pathological states. In our study, while the intervention group experienced an increase in the first 6 months and kept a steady concentration at 12 months, the control group had increased HDL cholesterol in the first 6 months which was slightly decreased at 12 months.

### Pro-inflammatory markers

High sensitivity C reactive protein (hs-CRP) is broadly used to monitor inflammatory processes, including autoimmune, infectious, tumoral, and metabolic diseases. Prospective epidemiological studies have reported elevated hs-CRP as an independent factor associated with cardiovascular events (26, 85). Dietary interventions usually lead to inflammatory profile improvement (86), we observed a reduction in hs-CRP levels across time in both groups, with no significant inter-group results.

Plasminogen activator inhibitor-1 plasma levels are positively associated with cardiovascular disease, thrombosis, fibrosis, and the progression of coronary syndromes (87). They are also positively correlated with individual risk factors (BMI, triglycerides, glucose, and mean arterial pressure) which may be indicative of their relevance in metabolic syndrome events (88). Diet composition has been demonstrated to affect circulating levels of PAI-1 and the fibrinolytic system as much as alcohol intake and smoking. High-fat diet consumption increases PAI-1 levels impairing clot lysis (29, 89). In our study, both groups produced a marked change in PAI-1 levels, although decreases were higher in the intensive group, mainly at the 12-month follow-up.

Cross-sectional studies have demonstrated that, compared to lean individuals, those with obesity have higher resistin levels (90–92). Some weight loss programs, however, have not always resulted in a decrease in circulating levels (31, 93, 94), while others reflect parallel reduction (95, 96). Regarding visfatin, weight loss programs have achieved a decrease in their levels, with no significant difference between them (94, 97). Nevertheless, there is evidence that a MedDiet has not always demonstrated an improvement in visfatin concentrations (98). In our study, resistin and visfatin levels displayed parallel behavior in both groups with an initial reduction at 6 months followed by steady maintenance at 12 months.

### Strengths and limitations

Our large sample size and randomized design provide high-quality evidence that minimizes confounding and bias influences. We have comprehensively assessed diverse cardiovascular risk biomarkers and satiety-related hormones. There are, however, some limitations. First, results were obtained in adult/elderly participants with metabolic syndrome and excess body weight; therefore, our findings cannot be extrapolated to other populations. Second, we observed only moderate differences between the two intervention arms. Such a finding was to be expected as the control group was an active comparator following a healthy traditional MedDiet. Moreover, due to the physiological regulation of ghrelin, among other hormones, the measurement of post-prandial levels would have been inestimable contribution, further research is warranted. Nevertheless, this randomized trial provides high-level evidence of the benefits of an intervention with a restrictive MedDiet and physical activity, especially on weight, waist circumference, leptin levels, lipid/glucose metabolism, blood pressure, and the pro-inflammatory marker PAI-1 at mid-and long-term intervention in subjects with metabolic syndrome. Given that such changes were maintained over time, and the marked palatability and acceptability of the MedDiet on the part of the consumers, MedDiet pattern interventions with hypocaloric diets could be a pertinent approach to weight loss.

## Data availability statement

The raw data supporting the conclusions of this article will be made available by the authors, without undue reservation.

## Ethics statement

The studies involving human participants were reviewed and approved by the Research Ethics Committees of all centers approved the study protocol during 2013 and 2014. The trial was registered in 2014 at (www.isrctn.com/ISRCTN89898870). The patients/participants provided their written informed consent to participate in this study.

## Author contributions

MF, JS-S, MM-G, DC, ER, FT, and RE designed the clinical trial. OC and MF designed the conceptualization sub-study. JH-R performed the formal and laboratory analysis. AT and JH-R carried out the statistical analysis. OC, MF, and JH-R drafted the manuscript. AT, DB, JS-S, MM-G, DC, RE, AG, OC, and MF revised and approved the final version. All authors contributed to the article and approved the submitted version.

## Abbreviations

MedDiet: Mediterranean diet
HbA1c: glycated hemoglobin
HDL cholesterol: high-density lipoprotein cholesterol
LDL cholesterol: low-density lipoprotein cholesterol
hs-CRP: highly sensitivity C-reactive protein
K2-EDTA: dipotassium ethylenediaminetetraacetic acid
GLP-1: glucagon-like peptide-1
PAI-1: plasminogen activator inhibitor-1
CV: coefficient of variation.
