# Supplementary material for: Mid- and long-term changes in satiety-related hormones, lipid and glucose metabolism, and inflammation after a Mediterranean diet intervention with the goal of losing weight: A randomized, clinical trial
Source: Front Nutr. 2022 Nov 18;9:950900. doi: 10.3389/fnut.2022.950900 (PMC9716624; doi:10.3389/fnut.2022.950900)
Supplement: Supplementary file 1 [file Data_Sheet_1.pdf]

Supplementary Material

Supplementary Material

1 Supplementary Figures

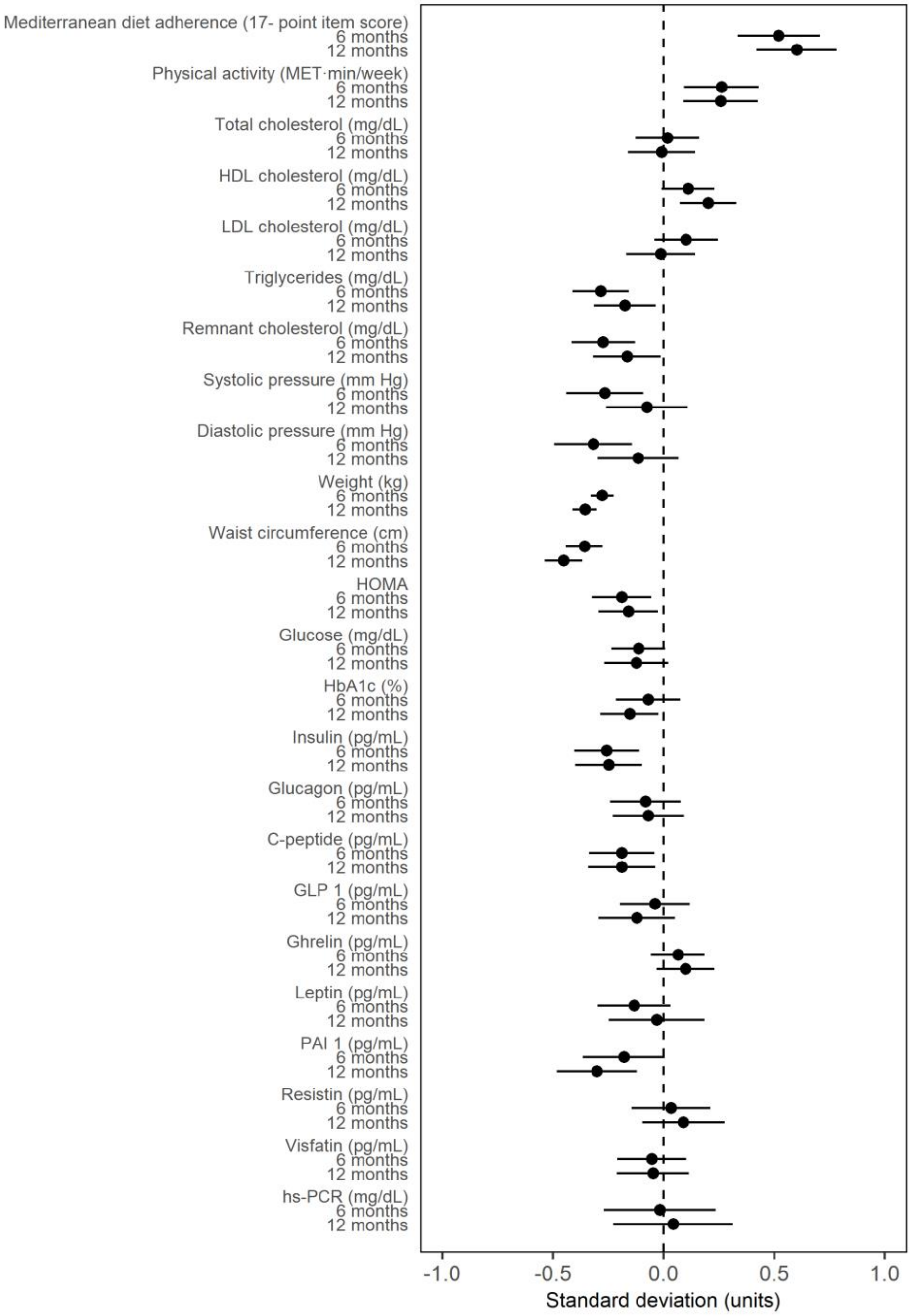

1.1 Supplementary Figure 1. Mean multivariable-adjusted differences (95% CI) for changes at 6- and 12-month follow-ups in 17-item questionnaire, physical activity, biomarkers and anthropometric measurements, lipid profile, carbohydrate metabolism, and hormones.

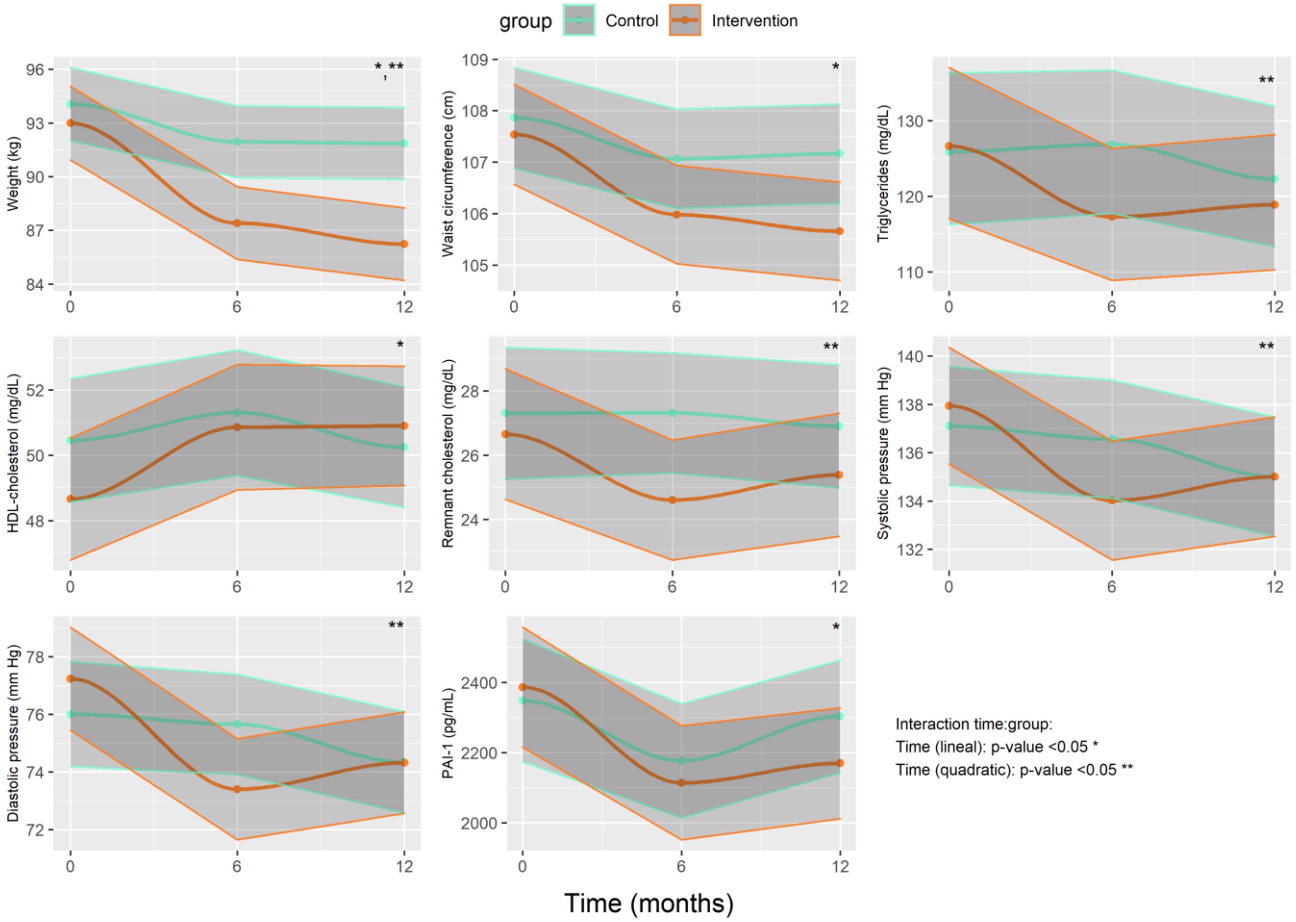

1.2 Supplementary Figure 2. Graphics of linear mixed-effect models in cardiovascular risk factors over time: weight, waist circumference, triglycerides, HDL-cholesterol, remnant cholesterol, systolic and diastolic blood pressure, and PAI-1.

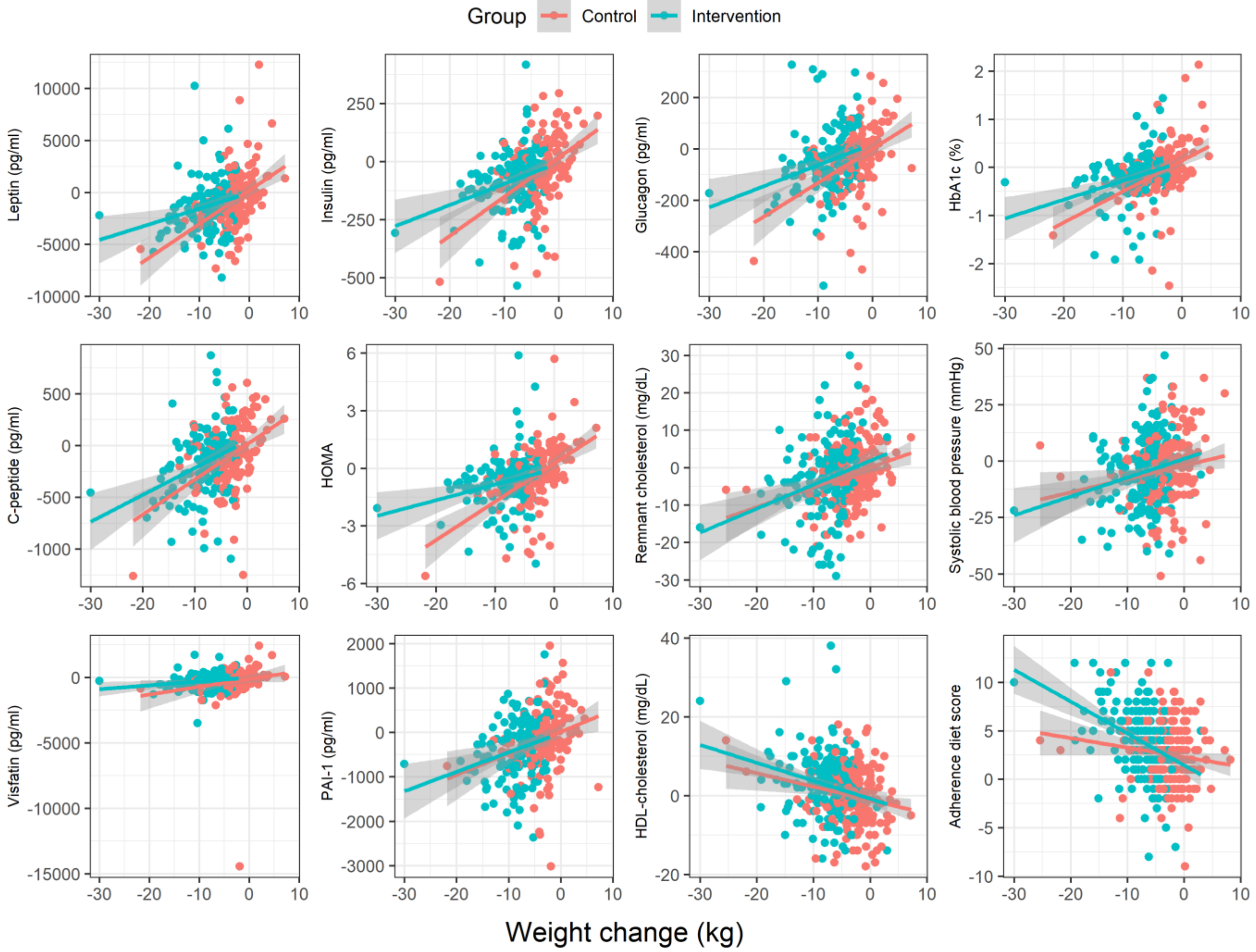

1.3 Supplementary Figure 3. Graphics of Pearson's correlation of weight loss with 12-months change of: leptin, insulin, glucagon, HbA1c, C-peptide, HOMA, remnant cholesterol, systolic blood pressure, visfatin and PAI-1, HDL cholesterol and adherence diet score.
